# Supplementary material for: Textured ferroelectric ceramics with high electromechanical coupling factors over a broad temperature range
Source: Nat Commun. 2021 Mar 3;12:1414. doi: 10.1038/s41467-021-21673-8 (PMC7977148; doi:10.1038/s41467-021-21673-8)
Supplement: Supplementary file 1 — Supplementary Information [file 41467_2021_21673_MOESM1_ESM.pdf]

Supplementary Information for

Textured Ferroelectric Ceramics with High Electromechanical Coupling Factors over a Broad Temperature Range

Shuai Yang[1], Jinglei Li[1]*, Yao Liu[1], Mingwen Wang[1], Liao Qiao[1], Xiangyu Gao[1], Yunfei Chang[2], Hongliang Du[1], Zhuo Xu[1], Shujun Zhang[3], Fei Li[1]*

[1] Electronic Materials Research Laboratory (Key Lab of Education Ministry), State Key Laboratory for Mechanical Behavior of Materials and School of Electronic Science and Engineering, Xi'an Jiaotong University, Xi'an, China.

[2] Condensed Matter Science and Technology Institute, School of Instrumentation Science and Engineering, Harbin Institute of Technology, Harbin, China.

[3] Institute for Superconducting and Electronic Materials, AIIM, University of Wollongong, Wollongong, NSW, Australia.

Supplementary Fig. 1 The length and thickness of BT templates. **a**, the SEM image of BT template; **b**, the distribution of length; **c**, the distribution of thickness.

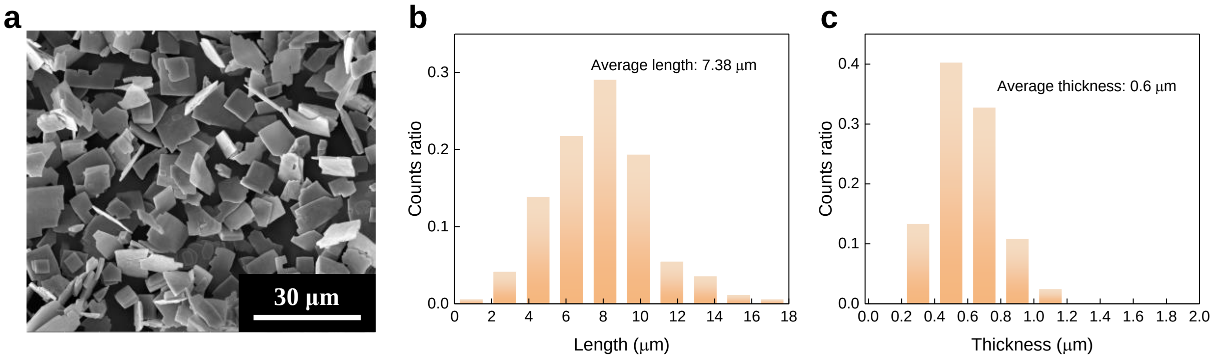

Supplementary Fig. 2 The SEM images and grain size distribution of random and textured 0.19PIN-0.445PSN-0.365PT ceramics with 3 vol.%, 5 vol.% and 7 vol.% BT templates (the large surface of the ceramics). Based on the SEM images, the average grain size is calculated by the Image Pro plus software.

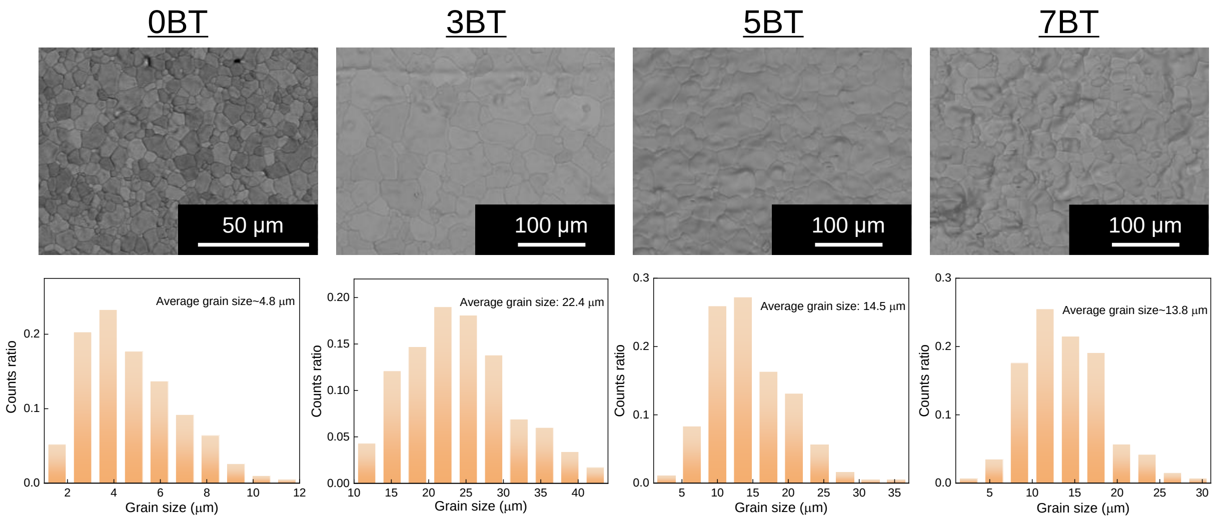

Supplementary Fig. 3 The temperature dependence of (222) diffraction pattern of nontextured 0.19PIN-0.445PSN-0.365PT ceramic.

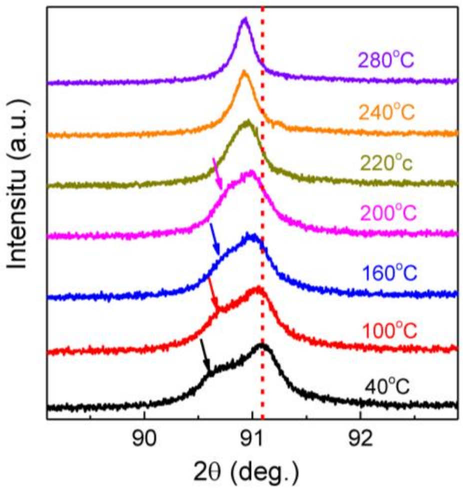

Supplementary Fig. 4 The temperature dependence of dielectric properties of x mol% Ba doped PMN-30PT random ceramic (x=0, 1 and 2).

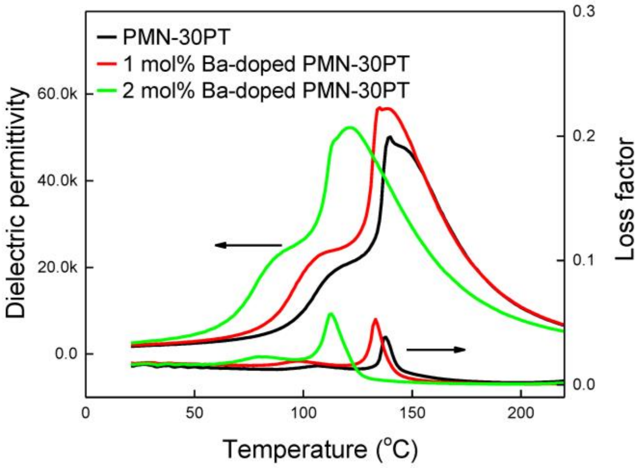

Supplementary Fig. 5 The SEM image and line scanning element analysis of EDS across BT and PIN-PSN-PT matrix in T-7BT sample. **a-b**, the SEM image and corresponding EDS analysis of a 'thick' template, whose thickness is about 0.5~1.2 μm, being within the thickness of the as-prepared templates; **c-d**, the SEM image and corresponding EDS analysis of a thin template. The EDS shows that for these 'thick' templates a small amount of Ba diffusion were observed in the ceramic matrix (Supplementary Fig. 5b). On the other hand, there are also some very thin templates in the textured ceramics, as shown in Supplementary Fig. 5c. The thickness is much smaller than the average thickness of the templates, being thought to be greatly reacted with the ceramic matrix. There are also some holes and fissions were observed around the template, being thought to be left after the reaction. The EDS results proves the significant diffusion of Ba from the thin template into the ceramic matrix (Supplementary Fig. 5d).

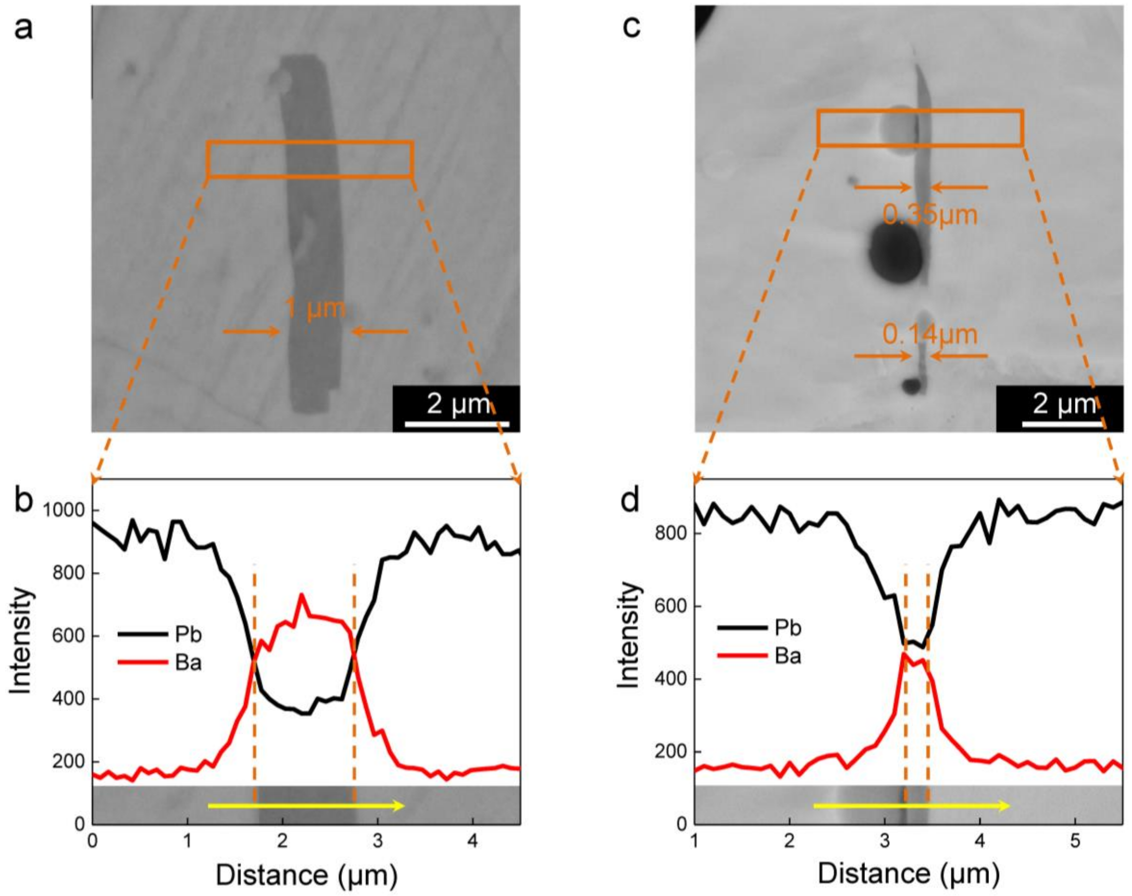

Supplementary Fig. 6 SEM image of PIN-PSN-PT textured ceramic with 3 vol.% BT templates.

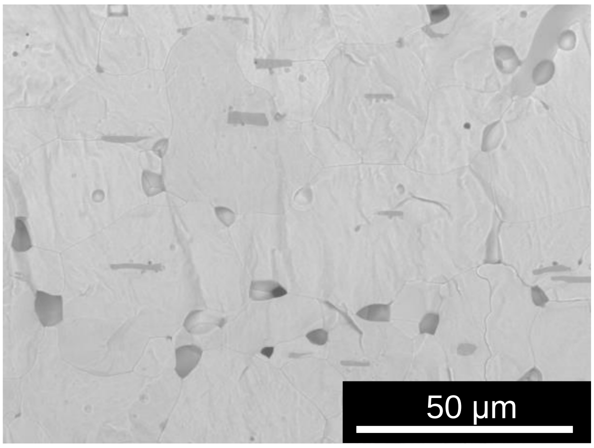

Supplementary Fig. 7 The <002> XRD rocking curves of PMN-0.28PT single crystal and textured PIN-PSN-PT ceramics. **a**, PMN-0.28PT single crystal; **b**, textured PIN-PSN-PT with 3 vol.% BT (T-3BT); **c**, textured PIN-PSN-PT with 5 vol.% BT (T-5BT); **d**, textured PIN-PSN-PT with 7 vol.% BT (T-7BT).

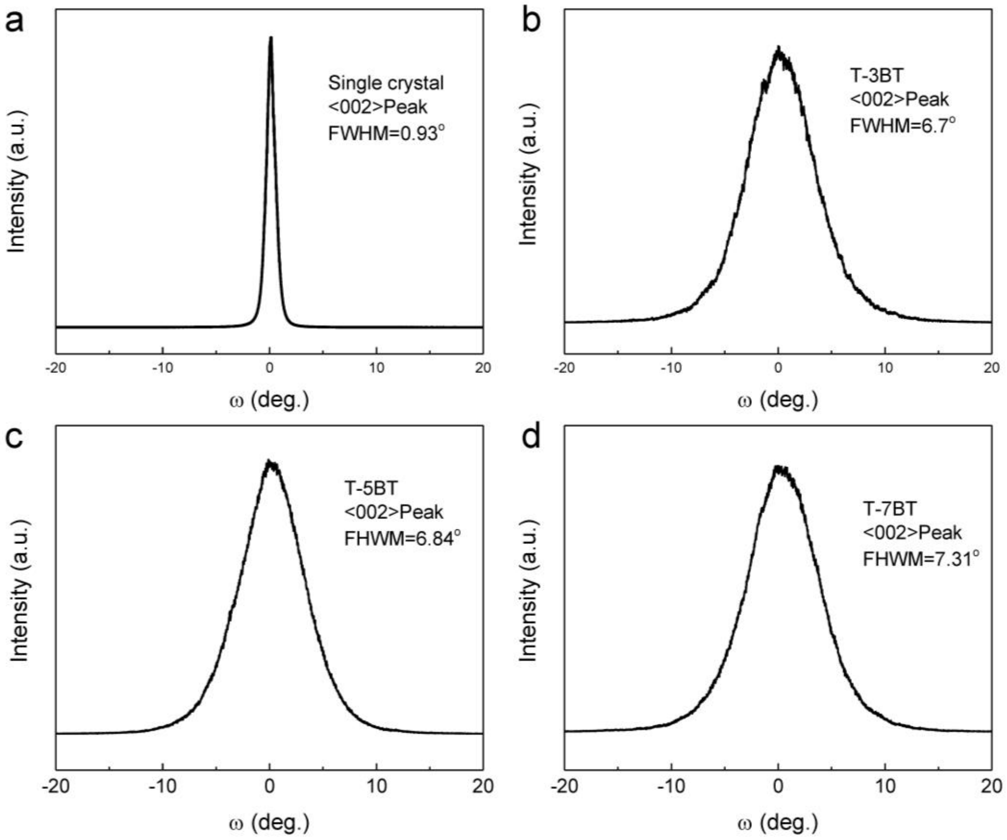

Supplementary Fig. 8 Electric-field-induced strains of T-3BT at various temperatures. The frequency and amplitude of the electric-field are 1 Hz and 20 kV cm^{-1}, respectively.

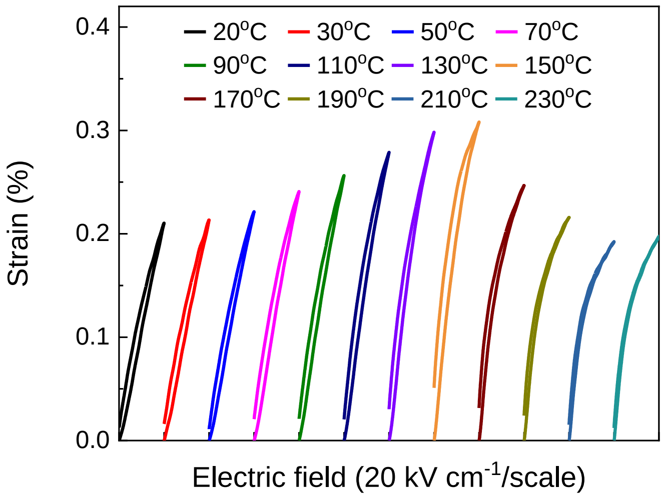

Supplementary Fig. 9 Grain orientation images of 0.19PIN-0.445PSN-0.365PT textured ceramic with 1 vol.% BT templates, measured by the SEM-EBSD technique.

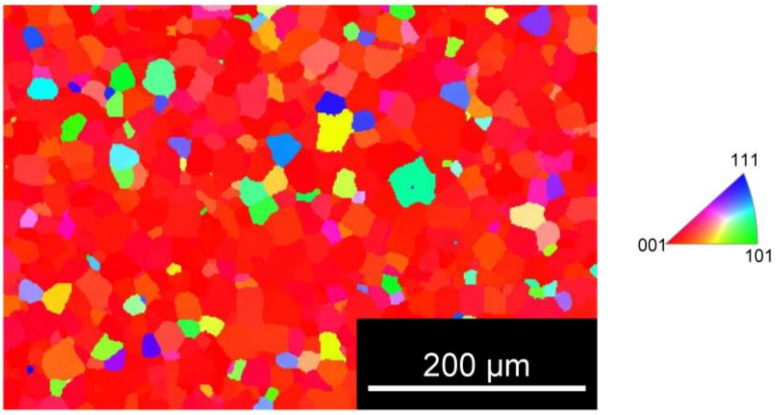

Supplementary Fig. 10 The SEM image and powder size distribution of PIN-PSN-PT. **a**, SEM image of PIN-PSN-PT powder; **b**, the distribution of powder size.

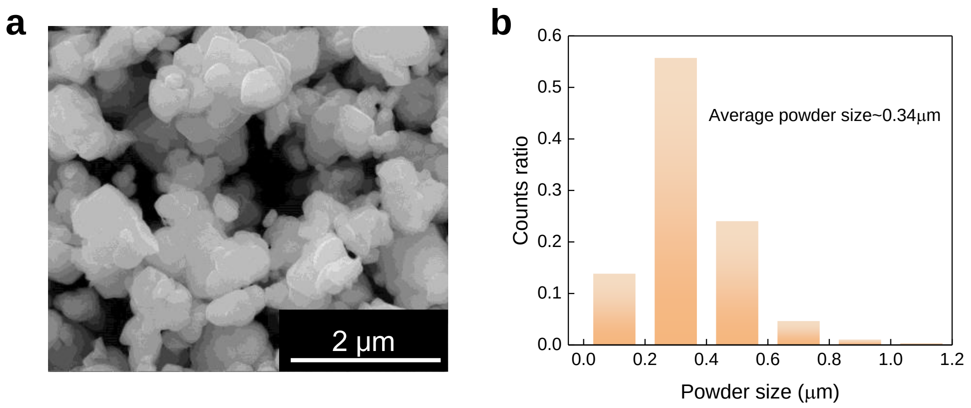

Supplementary Table 1 Densities of nontextured and textured 0.19PIN-0.445PSN-0.365PT ceramics. Five samples for each composition are used for the characterization of density.

	R-0BT	T-3BT	T-5BT	T-7BT
Theoretical density (g cm^{-3})	8.10	8.04	8.02	7.89
Measured density (g cm^{-3})	7.85±0.05	7.69±0.1	7.57±0.11	7.48±0.09
Relative density (%)	96.9±0.6	95.6±1.2	94.4±1.3	94.8±1.1

Supplementary Table 2 The parameters of textured 0.19PIN-0.445PSN-0.365PT ceramics for the calculation of k_p. The R-0BT, T-1BT, T-3BT, T-5BT and T-7BT indicate random, 1 vol.%, 3 vol.%, 5 vol.% and 7 vol.% BT textured 0.19PIN-0.445PSN-0.365PT ceramics, respectively.

Sample	f_r (kHz)	f_a (kHz)	f_{r1} (kHz)	σ	k^p (%)	k_p (%)
R-0BT	217.6	244.6	560.8	0.38	50.5	51.9
T-1BT	172.0	230.5	471.5	0.15	85.0	74.5
T-3BT	203.3	300	565.7	0.11	107.9	82.2
T-5BT	155.5	236	444.9	0.03	108.9	83.4
T-7BT	219.8	322.2	640.8	-0.02	95.2	80.5
